# Factor V Leiden (R506Q), Prothrombin G20210A, and MTHFR C677T Variants and Thrombophilia in Qatar Biobank Participants: A Case Control Study

**DOI:** 10.3390/pathophysiology31040044

**Published:** 2024-10-21

**Authors:** Sapha Shibeeb, Nada Al-Rayashi, Nehal Shams, Tameem Hadvan, Ejaife O. Agbani, Atiyeh M. Abdallah

**Affiliations:** 1 School of Health and Biomedical Sciences, RMIT University, P.O. Box 71, Bundoora, Melbourne, VIC 3083, Australia; sapha.shibeeb@rmit.edu.au; 2 Department of Biomedical Sciences, College of Health Sciences, QU Health, Qatar University, Doha 2713, Qatar; na1601159@student.qu.edu.qa (N.A.-R.); ns1701598@student.qu.edu.qa (N.S.); hadwan@qu.edu.qa (T.H.); 3 Department of Physiology and Pharmacology, Cumming School of Medicine, University of Calgary, Calgary, AB T2N 4N1, Canada; ejaife.agbani@ucalgary.ca

**Keywords:** thrombophilia, thrombosis, factor V Leiden, prothrombin, MTHFR, mean platelet volume, Qatar Biobank

## Abstract

**Background:** Thrombophilia, a predisposition to develop blood clots, is very common and can have serious sequelae. **Aim:** This study aimed to determine the prevalence of three thrombophilia-related genetic variants—factor V Leiden (FVL), prothrombin (F2) G20210A, and MTHFR C677T—in the Qatari population and their associations with self-reported thrombosis. **Methods:** We analysed samples from 408 Qatari participants [304 controls and 104 with self-reported thrombosis (deep vein thrombosis, pulmonary embolus, or ischaemic stroke)] from the Qatar Biobank. FVL (rs6025), F2 (rs1799963), and MTHFR (rs1801133) variants were genotyped using TaqMan assays. **Results:** Participants with self-reported thrombosis were older and more likely to be female. FVL A allele carriage (GA + AA vs. GG) was significantly higher in thrombosis cases (OR 3.6, *p* = 0.0002). In addition, individuals carrying FVL AA and GA genotypes had a lower mean platelet volume on average than those with the GG genotype (*p* = 0.03). MTHFR C677T did not show a similar association, and the F2 G20210A variant was too rare for analysis. **Conclusions:** There were significant differences in FVL A allele carriage between individuals with a history of thrombosis and the control group. Future research should explore the complex interplay between genetics and environment in thrombosis risk within this population.

## 1. Introduction

Thrombophilia is the general term describing an increased tendency for thrombus formation. Thromboembolic episodes result from an imbalance between thrombus formation and thrombus degradation and removal [1]. Thrombi contain platelets, fibrin, white and red blood cells, and neutrophil extracellular traps, with the composition varying depending on the disease or disease subtype [2]. Thrombotic events limit blood flow, and hence the supply of nutrients and oxygen to tissues, to cause ischaemic tissue damage and consequent clinical complications. The major arterial thrombosis sequelae are ischaemic heart disease (IHD), myocardial infarction (MI), and ischaemic stroke, while major venous thromboembolic (VTE) diseases include deep vein thrombosis (DVT) and pulmonary embolism (PE) [3].

Thrombosis-related disease is common, and its incidence varies across populations, with the incidence of VTE estimated to be 1–2 per 1000 individuals, IHD 15 per 1000 individuals, ischaemic stroke 1 per 1000 individuals, and MI 1.5 per 1000 individuals [4]. Overall, thromboembolic conditions account for 1 in every 4 deaths worldwide, and the WHO estimates that both arterial and venous thrombosis cases account for >10 million deaths annually [5]. Interestingly, worldwide public awareness about VTE (44–54%) is much lower than that for stroke and MI (85–88%) [6]. As elsewhere, the Arab population suffers from a variable but high incidence of thromboembolic disease. For example, in Saudi Arabia, the incidence of DVT was reported to be 2.7 per 1000 individuals in the Al-Madinah province [7], with a greater risk observed in females (58–65% of affected individuals) [7,8]. In Qatar, the incidence of VTE is just under 1 per 1000, but it has been reported that the incidence is increasing [9]. In critically ill patients, the incidence of VTE can reach as high as 50% [10,11]. For example, a study from Hamad Medical Corporation in Qatar reported an incidence of hepatic portal vein thrombosis of 0.5 per 1000, with a 30-day mortality rate of 19.5%: 71% in males and 29% in females [12].

Understanding the risk factors associated with thrombosis is crucial for the successful implementation of preventative strategies and public health interventions. Thrombosis is multifactorial, so both environmental and genetic factors contribute [13]. Advanced age is a significant risk factor for thrombosis, and the incidence increases sharply after 45 years of age due to changes in blood vessel integrity and reduced mobility [14]. Other risk factors include (i) obesity, due to increased circulating clotting factors and inflammation [15]; (ii) tobacco use and excessive alcohol consumption, by damaging blood vessels and promoting clot formation [16]; and (iii) medical comorbidities such as cancer, heart disease, autoimmune disorders, and inflammatory conditions. Data on the association between gender and thrombosis are inconsistent [4], and, while some studies have shown an association between ethnicity and VTE, with White individuals having lower incidence than African American and Asian individuals [17], these data are confounded by other factors and genetic predisposition.

Genetic factors play a crucial role in thrombosis development, increasing the risk of a first clot several-fold. Some genetic variants can lead to a prothrombogenic imbalance in the clotting system. Family and sibling studies of thrombosis genetics suggest 50–60% heritability [18,19], but identifying specific factors has remained a major challenge [20]. Some early important genetic factors were discovered using a candidate gene approach, with two types of variant reported: gain of function variants, such as factor V Leiden and prothrombin G20210A variants [21,22], and loss of function variants in the natural anticoagulants antithrombin, protein C, and protein S [23].

The factor V Leiden (FVL) variant is a single point substitution in nucleotide 1691 (exon 10) of the factor V (*F5*) gene, which results in an arginine to glutamine change at position 506, one of the activated protein C cleavage sites [24]. This protein is part of the coagulation downregulation pathway that proteolytically inactivates the non-enzymatic cofactors Va (FVa) and VIIIa (FVIIIa). Pathway dysfunction results in activated protein C resistance and reduced factor V susceptibility, leading to APC inactivation. The relative risk for VTE is increased approximately three- to eight-fold in factor V Leiden variant heterozygotes and the adjusted hazard ratio in heterozygotes compared with controls is 2.7 (95% confidence interval [CI] 1.8–3.8) [25]. The second major variant is in prothrombin or factor II, a vitamin K dependent protein [23]. G20210A is a point mutation in *F2* that leads to a guanine to adenine substitution at locus 20,210 [23]. Thrombin functions to activate factors V, VIII, and XIII and catalyse the conversion of fibrinogen to fibrin, the building block of the haemostatic plug. G20210A is located in the 3′UTR of the gene, and the mutation results in a 30% increase in prothrombin levels in heterozygous individuals and a 70% increase in homozygous individuals. The third major variant is in the methylenetetrahydrofolate reductase (*MTHFR*) gene, which encodes an enzyme that plays an important role in regulating the reduction of 5,10-methylenetetrahydrofolate to 5-methyltetrahydrofolate, which is responsible for homocysteine transformation to methionine. *MTHFR* is positioned on chromosome 1, and the C677T variant leads to an alanine to valine substitution at codon 222 and consequent hyperhomocysteinaemia. The presence of both FVL and the *MTHFR* variant further increases the risks of venous thromboembolism compared with the presence of FVL alone [26].

The consequences of thrombotic events include long-term disability, reduced quality of life, and increased healthcare costs. There is a striking lack of studies on genetic risk factors for thrombosis in Qatar, constituting a critical knowledge gap. As certain genetic variants increase the risk of thrombosis, quantifying the prevalence of these variants and understanding how they contribute to thrombosis among different ethnic groups can help in the development of targeted preventive strategies and personalised treatment options. The prevalence of these major thrombogenic variants is not known in the Qatari population. Therefore, here, we studied the prevalence of the FVL (rs6025), *F2* (rs1799963), and *MTHFR* (rs1801133) variants in the Qatari population. Our second aim was to investigate the association between these variants and a history of thrombosis in the Qatari population. To achieve this, we exploited access to 408 participants from the Qatar Biobank (QBB), which provides biological samples coupled with excellent clinical annotation, including self-reported thrombotic events.

## 2. Materials and Methods

### 2.1. Ethical Approvals and Study Participants

This was a case control genetic association study of Qatar Biobank (QBB) participants who self-reported a history of blood clots. The study participants were identified in the QBB. The Institutional Review Boards of Qatar University (protocol # QU-IRB 1905-E/23) and the QBB (protocol # QF-QBB-RES-ACC-00062-0195) approved this study. All experiments were performed according to approved guidelines. All participants were 18 years old and above, and no exclusion criteria were applied. The QBB recruited male and female Qatari nationals or long-term residents (living in Qatar for >15 years) aged between 18 and 70 years. QBB obtained written informed consent from all participants. Sociodemographic information was collected by a self-administered questionnaire. Disease history, medication use, and other clinical data such as body weight, height, and blood pressure were collected by interview with a specialised nurse. Samples and data were analysed from 408 Qatari participants (304 controls and 104 with self-reported thrombotic events).

### 2.2. Definition of Thrombosis

Information about prior thrombotic events was collected by asking participants “Has a doctor ever told you that you have had blood clot?” (Yes, No, I’m not sure); if Yes, “What types of blood clot were you diagnosed with: blood clot in the arms or legs (DVT), blood clot in the lung (PE), blood clot in the brain (ischaemic stroke)?” Participants were given the choice to select more than one answer.

### 2.3. Genotyping Techniques

A NanoDrop spectrophotometer (Thermo Fisher Scientific, Waltham, MA, USA) was used to quantify all DNA samples. Then, samples were stored at −20 °C until use. The duplex quantitative TaqMan 5′ allelic discrimination assay (Applied Biosystems, Foster City, CA, USA) was used for genotyping experiments, following the manufacturer’s instructions. The factor V Leiden variant (A506G, rs6025; assay ID: C_11975250_10), the prothrombin (F2) variant (G20210A, rs1799963; assay ID: C_8726802_20), and the MTHFR variant (C677T, rs1801133; assay ID: C_1202883_20) were examined. The TaqMan 5′ allelic discrimination assay included TaqMan genotyping master mix, 40x SNP genotyping assay mix, DNase-free water, and 20 ng DNA in a final volume of 10 µL. PCR reactions were performed using a StepOne or StepOnePlus real-time PCR system. The amplification protocol was 95 °C for 3 s followed by 40 cycles at 95 °C for 0.03 s and 60 °C for 60 s (annealing/extension). Fluorescence detection was conducted at 60 °C. Non-template negative controls were included in each run. For quality control, 10% of randomly selected samples were repeated to confirm the genotype.

### 2.4. Statistical Analysis

Deviation from the Hardy–Weinberg equilibrium (HWE) was checked for all genotyping data using chi-square contingency table analysis. Statistical analyses were conducted using SPSS v28 (IBM Statistics, Armonk, NY, USA) and confirmed using a freely available online statistical tool, VassarStats (www.vassarstats.net, accessed on 6 September 2024). The unpaired Student’s *t*-test was used to compare the mean and standard deviation (SD) between groups. Genotype and allele frequencies were determined by direct counting, and chi-square contingency table analysis or Fisher’s exact test, as appropriate, were used to test for any significant differences in the distributions of genetic variants between cases and controls. Linear regression analysis was used to test for any association between the genetic variants and any other parameters. Odds ratios (ORs) and confidence intervals (95% CIs) were used to determine the strength of the results. Any *p*-value < 0.05 was deemed statistically significant.

## 3. Results

### 3.1. Cohort Characteristics

The baseline characteristics of the cohort are presented in Table 1. In total, 408 QBB participants (304 controls and 104 cases) were included in this study. There was an equal distribution of males and females overall, but there were more females than males in the cases group (*p* < 0.0001). Among 104 individuals who reported to have been diagnosed with blood clot, 67% reported DVT; 18% reported PE; 15% reported DVT and PE; and none reported ischaemic stroke.

### 3.2. Distribution of FV Leiden Variant

The FVL G1691A variant genotypes, allele frequencies, and allele carriage distributions are shown in Table 2. Control and case cohorts did not deviate from HWE. There was a significant difference in the distribution of FVL variant genotype frequency between cases and controls (χ^2^ = 15.9, *p* = 0.0004). The A allele frequency was higher in cases than controls (χ^2^ = 16.5, *p* = 0.0001, OR 3.6, 95% CIs 1.9–6.8). The A allele carriage (GA + AA vs. GG) was associated with an increased risk of thrombosis (χ^2^ = 14.3, *p* = 0.0002, OR 3.6, 95% CIs 1.8–7.0) (Table 2).

### 3.3. Distribution of the MTHFR Variant

*MTHFR* C677T variant genotypes, allele frequencies, and allele carriage distributions are shown in Table 3. Control and case cohorts did not deviate from HWE. There was no significant difference in the distribution of the *MTHFR* C677T variant genotype, allele frequency, and allele carriage between cases and controls (Table 3).

### 3.4. Distribution of the F2 (Prothrombin) Variant

The presence of the *F2* (prothrombin) G20210A variant in the population is presented in Table 4. The variant was very rare in the control group, and none of the case group carried the rare allele (A).

### 3.5. FV Leiden Variant and Laboratory Parameters

**Figure 1:**
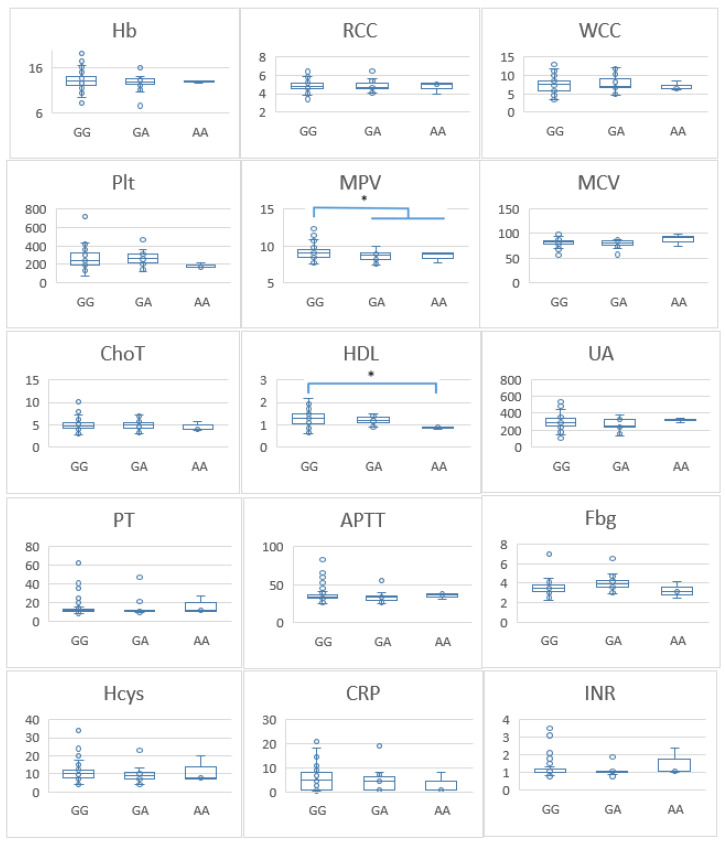
Differences in laboratory parameters between patients with different factor V genotypes. Hb—haemoglobin (g/dL); RCC—red blood cell counts; WCC—white blood cell counts; PLT—platelet counts; MPV—mean platelet volume (fl); MCV—mean corpuscular volume (fl); ChoT—total cholesterol (mmol/L); HDL—high-density lipoprotein cholesterol (mmol/L); UA—uric acid (µmol/L); PT—prothrombin time (seconds); APTT—activated partial thromboplastin time (seconds); Fbg—fibrinogen (g/L); Hcys—homocysteine (µmol/L); CRP—C-reactive protein (mg/L); INR—international normalised ratio. * *p*-value ≤ 0.05 considered significant. * Significant differences were found between two laboratory test (MPV and HDL) and FV G1691A variants in this cohort of patients.

**Figure 2:**
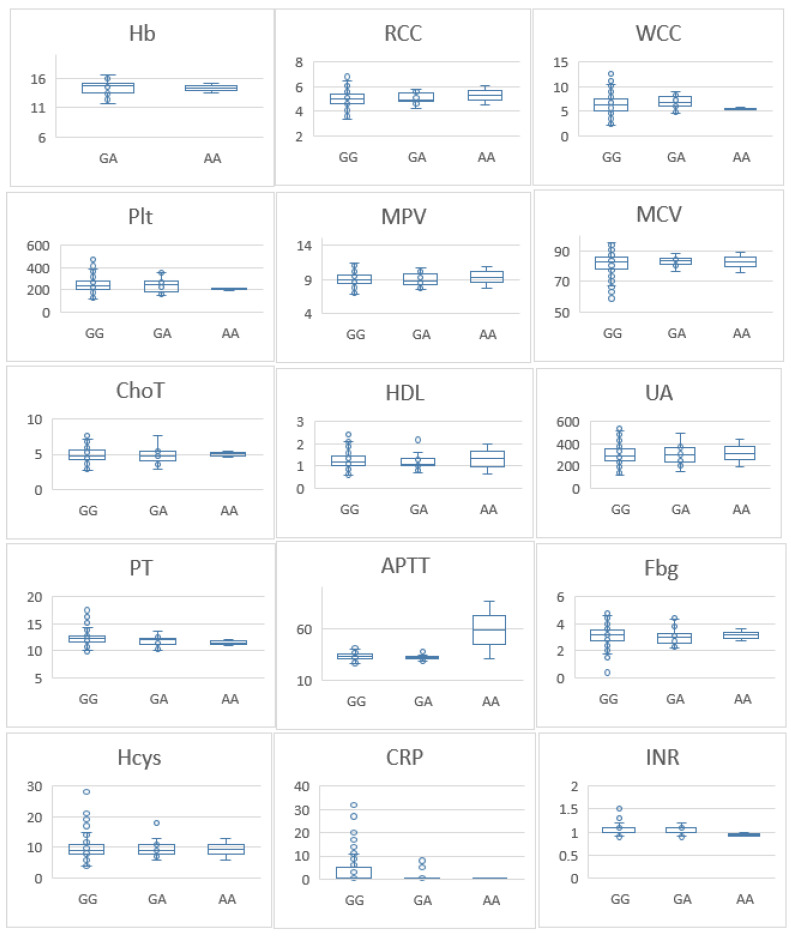
Differences in laboratory parameters among healthy controls with different Factor V genotypes. Hb—haemoglobin (g/dL); RCC—red blood cell counts; WCC—white blood cell counts; PLT—platelet counts; MPV—mean platelet volume (fl); MCV—mean corpuscular volume (fl); ChoT—total cholesterol (mmol/L); HDL—high-density lipoprotein cholesterol (mmol/L); UA—uric acid (µmol/L); PT—prothrombin time (seconds); APTT—activated partial thromboplastin time (seconds); Fbg—fibrinogen (g/L); Hcys—homocysteine (µmol/L); CRP—C-reactive protein (mg/L); INR—international normalised ratio. No significant difference was found between FV G1691A variants and healthy individuals in this cohort.

To further investigate the association between the FVL variant and thrombogenic mechanisms, we compared haemoglobin (Hb) levels, red blood cell counts (RCC), white blood cell counts (WCC), platelet counts (PLT), mean platelet volume (MPV), mean corpuscular volume (MCV), total cholesterol (ChoT), high-density lipoprotein cholesterol (HDL), uric acid (UA), prothrombin time (PT), activated partial thromboplastin time (APTT), fibrinogen (Fbg), homocysteine (Hcys), C-reactive protein (CRP), and international normalised ratio (INR) between genotypes. Individuals carrying GA and AA genotypes had lower MPV than those with the GG genotype (*p* = 0.03). Additionally, those with the AA genotype had lower HDL levels compared with individuals with the GG genotype (*p* ≤ 0.05) (Figure 1). No significant differences were observed for the other laboratory parameters between the three genotypes. We conducted a parallel analysis for the control group, which revealed no significant differences in laboratory parameters (Figure 2).

## 4. Discussion

In this study, our primary objective was to assess the prevalence of genetic factors predisposing to thrombophilia within the Qatari population by exploiting a case control design, where cases had a self-reported history of thrombosis. Our results demonstrated that participants with previous thrombosis were older and more likely to be female. Furthermore, individuals with a history of thrombosis exhibited statistically significant higher levels of plasma fibrinogen and homocysteine, consistent with previous research showing that individuals with a history of thrombosis exhibit increased levels of both homocysteine and fibrinogen [27,28]. In our cohort, A allele frequency of the *F5* G1691A variant was 3% and the genotype frequency (GA) was 6% in the healthy control cohort. For *MTHFR* C677T, the T allele frequency was 19% and the genotype frequency (GA) was 33% in the healthy controls. Finally, the A allele frequency for the *F2* G20210A was 1% and the genotype frequency (GA) was also 1% in the healthy control cohort. The genotype, allele, and allelic carriage frequencies of the *F5* G1691A variant were associated were significantly different between cases and controls. However, the *MTHFR* C677T variant was not associated with thrombosis in our population. We could not perform statistical analysis for *F5* G20210A as it was very rare. Finally, we found that individuals carrying GA and AA *F5* G1691A genotypes had lower MPVs than those with the GG genotype. However, in the control group, this association was not detected.

The prevalence of the *F5* G1691A variant has been reported in numerous studies from the Arab region and internationally. The prevalence of the *F5* G1691A A allele variant varies widely in different Arab countries, e.g., Palestinians (11%), Jordanians (9%), Lebanese (8%), Syrians (5%), Bahrainis (4%), Tunisians (3.5%), Kuwaitis (2.2%), and Saudi Arabians (1%) [26]. Interestingly, the Qatari population had an A allele frequency of 3%, which is an intermediate prevalence in Arab populations. The prevalence of the *F5* G1691A A allele variant in our patient cohort was 11%, emphasising the importance of factor V Leiden as a risk factor for thrombosis in our population.

To our knowledge, this is the first time that the prevalence and importance of this variant have been reported in the Qatari population. Our findings are consistent with other studies from the region and worldwide. In a recent meta-analysis, the risk of recurrent VTE was increased 46% in patients with the heterozygous *F5* G1691A variant [24]. Interestingly, women with the *F5* G1691A variant are at increased risk of VTE, particularly in those diagnosed with obesity, hypertension, chronic kidney disease, or dyslipidaemia [29]. Moreover, the *F5* G1691A variant has a different role in different diseases. The association between the *F5* G1691A variant and recurrent pregnancy loss was analysed in a meta-analysis, which found that, in Asian and African populations but not European and American populations, the *F5* G1691A variant was associated with recurrent pregnancy loss [30]. Another meta-analysis confirmed the relationship between the FV G1691A variant and ischaemic strokes in young adults [31]. This analysis not only confirms the association between this genetic variant and ischaemic strokes but also underscores the potential for significant advances in healthcare quality through routine testing.

*F5* G1691A is a single nucleotide change that changes the G (guanine) to an A (adenine) at the 1691 position in exon 10, consequently causing an amino acid substitution (arginine to glutamine, R506Q) [32]. Factor V is a procoagulant protein that accelerates the conversion of prothrombin to thrombin, and it is inactivated by activated protein C. The FVL variant is a gain-of-function variant that causes resistance to activated protein C, thereby predisposing individuals to thrombosis [13]. Molecular testing for the *F5* G1691A variant and other thrombotic variants is important not only to obtain a diagnosis but to also offer valuable insights into predicting and potentially preventing future thromboembolic events. This is also true for “normal” individuals, irrespective of their prior history of thromboembolic disease [33]. Recognising the importance of this, the American College of Medical Genetics (ACMG) has established stringent standards for laboratory testing for FVL, emphasising the necessity of genetic testing [34]. Specifically, genetic testing is recommended for individuals with a familial predisposition to FVL, families with a documented history of thrombophilia, patients who have received a diagnosis of antiphospholipid syndrome, or individuals requiring anticoagulant therapy. In cases where individuals test positive in a functional assay for APC resistance, genetic testing becomes not only recommended but crucial for confirming the diagnosis and guiding appropriate management strategies [35]. Routine testing for FVL or other thrombotic variants is not typically conducted in healthy individuals as part of standard medical practice. Therefore, the question that arises is, considering the substantial global burden of morbidity and mortality attributed to thrombotic events, is there merit in exploring the landscape of inherited thrombophilia even in seemingly healthy subjects? This issue has sparked some academic and clinical controversy. The fact that the FVL G1691A variant is present in 1–10% of otherwise healthy individuals, the majority of whom do not develop thrombosis, has led to the consensus that exposing carriers to anticoagulant therapy may introduce a significant risk of adverse outcomes, including serious bleeding, so there has been no strong support for routine testing for FVL or other thrombotic variants [36].

We also found that, in individuals with a history of thrombosis, FVL G1691A allelic A carriage (GA and AA genotypes) had a lower MPV than those with the GG genotype. This association was not present in the control group. MPV is a measure of the size of platelets, and it has been suggested that large platelets can increase the risk of thrombosis. However, this is controversial. In a recent Mendelian randomisation study, a higher MPV was associated with a significantly lower risk of DVT [37]. In addition, low MPV is associated with many high-grade inflammatory diseases and thrombosis in those diseases [38]. The effect of the size of the platelets in thrombosis is still controversial, and our results indicate that small platelets may enhance the chance of developing thrombosis in the presence of FVL.

MTHFR directs folate species either to DNA synthesis or to homocysteine re-methylation. A number of genetic variants have been reported in the *MTHFR* gene, but the C677T mutation (rs1801133) is the most studied and clinically important, as it reduces enzyme activity [39]. The *MTHFR* C677T variant has been reported as a risk factor for several disorders including cardiovascular diseases, congenital heart defects, and type 2 diabetes mellitus [40,41,42]. The association between the *MTHFR* C677T variant and thrombosis is controversial. In a large meta-analysis of 120,000 cases and 180,000 controls, *MTHFR* C677T was associated with thrombosis in Chinese/Thai populations but not in other populations such as White and Black African individuals [43]. In a recent study, carriers of the homozygous TT genotype developed idiopathic portal vein thrombosis 20 years earlier than wild-type carriers [44]. The study found that individuals with the *MTHFR* TT genotype were at greater risk of thrombosis if they also had high homocysteine levels [44], and this association between a moderate increase in homocysteine has been reported previously [45,46]. These interesting findings highlight the importance of analysing associations of the *MTHFR* variant in the presence of other important factors that influence thrombosis and other cardiovascular diseases [13,47]. Although we found that our participants with a history of previous thrombosis had significantly higher blood homocysteine levels, only one individual with the *MTHFR* TT genotype had a high homocysteine level. However, formal analysis between homocysteine levels and specific genotypes was not possible due to the cohort size

The PT G20210A mutation increases prothrombin levels through increased prothrombin synthesis. Our study revealed a very low frequency (1%) of the PT G20210A heterozygous (GA) genotype in the control group and zero percent in the thrombosis group. Furthermore, we did not detect the homozygous (AA) genotype in either of the control and thrombosis groups. The prevalence rates in our participants were comparable to those in other Arab populations such as Saudi Arabians (GA, 0% and AA, 0.0%) and Bahrainis (GA, 1% and AA, 0%) [26].

A major limitation of our study is the small sample size. In addition, this study was based on self-reported thrombotic events, which may have been subject to recall bias or misunderstanding. We did not account for other confounding factors that influence the relationship between the genetic variants and thrombophilia such as lifestyle factors, comorbidities, or medication usage. In addition, genetic diversity within the Qatari population, influenced by ancestry or other factors, could introduce population stratification which could lead to spurious findings. The finding of lower MPV in FVL AA and GA carriers is intriguing, but it could be influenced by other factors not accounted for in this study, such as platelet count, medication use, or underlying health conditions. Finally, we performed multiple hypothesis testing, which might compromise the validity of our findings.

## 5. Conclusions

This study revealed significant differences in FVL A allele carriage between individuals with a history of thrombosis and the control group. Participants with previous thrombotic episodes tended to be older and predominantly female, and they exhibited statistically significant higher levels of plasma fibrinogen and homocysteine, consistent with previous reports. We detected a significant association between thrombosis and the presence of FVL in the general Qatari population. Furthermore, the *MTHFR* C677T variant did not show a statistically significant association with thrombosis and the PT G20210A variant was very rare in our population. Further research is needed to elucidate the complex interplay between genetic and environmental factors in thrombosis risk within this population.

## Figures and Tables

**Table 1:** Baseline clinical and general characteristics of the study participants.

Variable	Total (n = 408) *	Cases (n = 104) *	Controls (n = 304) *	*p-*Value ^†^
Sex				
Male (n%)	201 (49.3%)	34 (32.7%)	167 (54.9%)	<0.0001
Female (n%)	207 (50.7%)	70 (67.3%)	137 (45.1%)	
Age (years)	36.6 ± 11.9	46.6 ± 12.6	33.2 ± 9.5	<0.0001
Hb (g/dL)	13.7 ± 1.8	13.1 ± 1.8	13.8 ± 1.8	0.007
RCC (×10^6^/µL)	4.98 ± 0.56	4.90 ± 0.63	5.00 ± 0.56	0.13
WCC (×10^3^/µL)	6.71 ± 1.95	7.47 ± 2.18	6.49 ± 1.71	<0.0001
PLT (×10^3^/µL)	248 ± 69	257 ± 93	246 ± 61	0.17
MPV (Fl)	9.05 ± 0.94	9.02 ± 0.87	9.05 ± 0.96	0.8
MCV (fl)	81.6 ± 7.06	81.6 ± 7.9	81.8 ± 6.74	0.8
Total cholesterol (mmol/L)	4.92 ± 1.0	4.9 ± 1.17	4.9 ± 0.94	1
HDL (mmol/L)	1.27 ± 0.35	1.29 ± 0.35	1.26 ± 0.34	0.4
Uric acid (µmol/L)	300.7 ± 81.3	289.3 ± 77	304.6 ± 82.4	0.01
PT (s)	12.8 ± 4.2	14.7 ± 8.3	12.1 ± 1.1	<0.0001
INR	1.1 ± 0.39	1.22 ± 0.51	1.04 ± 0.095	<0.0001
APTT (s)	33.9 ± 5.7	35.8 ± 9.1	33.9 ± 4.1	0.004
Fib (g/L)	3.27 ± 0.68	3.61 ± 0.72	3.15 ± 0.63	<0.0001
Homocysteine (µmol/L)	9.7 ± 3.12	10.47.3 ± 4.52	9.45 ± 2.76	0.007
CRP (mg/L)	3.6 ± 4.9	5.3 ± 5.2	2.9 ± 4.65	<0.0001

* Data presented as mean and standard deviation (M ± SD). ^†^ Comparing cases and controls. *p* ≤ 0.05 considered significant. Abbreviations: Hb—haemoglobin, RCC—red cell count, WCC—white cell count, PLT—platelet count, MPV—mean platelet volume, PT—prothrombin time, APTT—activated partial thromboplastin time, Fib—fibrinogen, n—number, M ± SD—mean ± standard deviation.

**Table 2:** Genotype, allele frequencies, and allele carriage for factor V Leiden variant in case and control populations.

FV G1691A (rs6025)	Control (N = 304)		Cases (N = 104)		*X^2^*	*p*-Value	OR (95%CI)
	Count	Frequency	Count	Frequency			
GG	285	0.937	84	0.81	15.9	**0.0004**	
GA	18	0.06	17	0.16			
AA	1	0.003	3	0.03			
G	588	0.97	185	0.89	16.5	**0.0001**	3.6 (1.9–6.8)
A	20	0.03	23	0.11			
GG + GA	303	0.997	101	0.97	5.5	0.053	
AA	1	0.003	3	0.03			
GA + AA	19	0.063	20	0.19	14.3	**0.0002**	3.6 (1.8–7.0)
GG	285	0.937	84	0.81			

Significant values are shown in bold. Abbreviations: FV—factor V, N—number of cases and controls. *p* ≤ 0.05 considered significant. The genotype frequency, allele frequency, and allelic carriage for the FV G1691A were found to be associated with thrombosis in the Qatari population.

**Table 3:** Genotype, allele frequencies, and allele carriage for the MTHFR variant in cases with self-reported thrombosis and the control population.

*MTHFR* C677T (rs1801133)	Control (N = 304)		Cases (N = 104)			
	Count	Frequency	Count	Frequency	*X^2^*	*p*-Value
CC	195	0.64	63	0.61	2.97	0.23
CT	100	0.33	34	0.33		
TT	9	0.03	7	0.07		
C	490	0.81	160	0.77	1.07	0.3
T	118	0.19	48	0.23		
CC + CT	295	0.97	97	0.93	2.01	0.15
TT	9	0.03	7	0.07		
CT + TT	109	0.36	41	0.39	0.28	0.59
CC	195	0.64	63	0.61		

Abbreviations: N—number of cases and controls, MTHFR—methylenetetrahydrofolate reductase. No association between thrombosis and MTHFR C677T was found in this patient cohort.

**Table 4:** Genotype, allele frequencies, and allele carriage for the *F2* variant in cases with self-reported thrombosis and the control population.

*F2* (Prothrombin) G20210A (rs1799963)	Control (N = 318)		Cases (N = 104)	
	Count	Frequency	Count	Frequency
GG	300	0.99	104	1
GA	4	0.01	0	0
AA	0	0	0	0
G	604	0.99	208	1
A	4	0.01	0	0
GG + GA	304	1	104	1
AA	0	0	0	0
GA + AA	4	0.01	0	0
GG	300	0.99	104	1

Abbreviations: *F2*—Factor II, N—number of cases and controls.

## Data Availability

Data were obtained from Qatar Biobank (https://www.qatarbiobank.org.qa/) on 15 August 2022. Data are available from Qatar Biobank upon request.
